# *Fusarium napiforme* systemic infection: case report with molecular characterization and antifungal susceptibility tests

**DOI:** 10.1186/2193-1801-3-492

**Published:** 2014-08-30

**Authors:** Marcela de Souza, Tetsuhiro Matsuzawa, Luzia Lyra, Ariane Fidelis Busso-Lopes, Tohru Gonoi, Angélica Zaninele Schreiber, Katsuhiko Kamei, Maria Luiza Moretti, Plínio Trabasso

**Affiliations:** Department of Internal Medicine, School of Medicine, University of Campinas, Campinas, São Paulo Brazil; Medical Mycology Research Center, Chiba University, Chiba, Japan; Department of Clinical Pathology, School of Medicine, University of Campinas, Campinas, São Paulo Brazil; LIM 46 – Laboratory of Parasitology – HC/FMUSP, Kragujevac, São Paulo Brazil

**Keywords:** Fusariosis, Opportunistic Infections, Immunocompromised host

## Abstract

**Introduction:**

During the last decades, *Fusarium* spp. has been reported as a significant cause of disease in humans, especially in immunocompromised patients, who have high risk of invasive life-threatening disease. Fusarium species usually reported as cause of human disease are *F. solani*, *F. oxysporum* and *F. verticillioides*.

**Case description:**

We describe the second case in the literature of disseminated fusariosis caused by *Fusarium napiforme*, that occurred in a 60-year-old woman with multiple myeloma after subsequent cycles of chemotherapy.

**Discussion and Evaluation:**

We identified the *F. napiforme* not only by standard morphologic criteria by macroscopic and microscopic characteristics, but also confirmed by molecular biology methods, including sequencing. The antifungal susceptibility of the *F. napiforme* isolates were tested to seven antifungal drugs; the azoles were the most active drug against all the isolates tested.

**Conclusions:**

*Fusarium* spp. are of relevance in medical mycology, and their profiles of low susceptibility to antifungal drugs highlight the importance for faster and more accurate diagnostic tests, what can contribute to an earlier and precise diagnosis and treatment.

## Background

*Fusarium* are widely distributed fungi in soil, plants, plant debris and other organic substrates, and in water systems. During the last decades, *Fusarium* spp. has been reported as a significant cause of disease in humans, especially immunocompromised patients (Jureen et al. 2008; Bourgeois et al. 2010; De Pinho et al. 2012; Calcaterra et al. 2013).

In immunocompetent persons, *Fusarium* usually causes localized infections (Bourgeois et al. 2010; De Pinho et al. 2012; Calcaterra et al. 2013; Homa et al. 2013). Conversely, immunocompromised hosts, mainly those with acute onco-hematological diseases or after allogeneic hematopoietic stem cell transplant, have high risk of invasive life-threatening diseases. In such patients, invasive fusariosis (IF) is relatively resistant to standard antifungal therapy limiting their treatment options (Scheel et al. 2013; Pereira et al. 2013).

*Fusarium* species reported as cause of human disease and *F. solani*, *F oxysporum* and *F. verticillioides* are the most frequently species causing IF (Gupta et al. 2000; Tezcan et al. 2009).

The first case of disseminated fusariosis was described in 1973 (Cho et al. 1973). Since then, there was a significant increase in the occurrence of disseminated disease, probably reflecting the increase in number of immunocompromised hosts (Bourgeois et al. 2010). There are reports of IF worldwide (Martino et al. 1994; Nucci and Anaissie 2007; Tortorano et al. 2008; Slavin et al. 2012; Nucci et al. 2013). The first case of disseminated fusariosis due to *F. napiforme* was described in 1993 (Melcher et al. 1993) but, there were no subsequent reports of disseminated disease. Thus, to our knowledge, we describe the second case of the literature of disseminated fusariosis caused by *F.napiforme*, and the first report of *F. napiforme* confirmed by molecular biology methods, including sequencing.

## Case description and molecular identification of the clinical isolates

### Case description

A 60-year-old woman was diagnosed with stage IIIB multiple myeloma (MM) in 2005. She was treated with chemotherapy, 6 cycles of VAD (vincristine + doxorubicin + dexamethasone) in 2006, then cyclophosphamide in 2007. This treatment was followed by an autologous bone marrow transplant in 2008. The disease relapsed in October 2010. She underwent decompressing lumbar spine surgery in 2010 and received various courses of treatment with zoledronic acid throughout her follow-up. The patient had also radiotherapy in 2009 (spine) and 2011 (spleen), followed by salvage chemotherapy throughout 2011 (cyclophosphamide + thalidomide + dexamethasone-CTD, then velcade + thalidomide + dexamethasone-VTD, and finally cyclophosphamide + prednisone). At this point she was considered to present a very good partial response (VGPR). In October 2012, during a routine medical consultation, she complained of palpitation and dyspnea starting 10 days before. The patient presented with hypercalcemia and had to be admitted for the treatment of this condition. During the admission, a second BMT was proposed to treat the refractory MM; therefore, myelosuppressive chemotherapy was initiated. After one week the neutrophil count fell to zero and painful vasculitis lesions arose in lower abdominal region and in the left thigh. Blood cultures were withdrawn and skin biopsy was performed. Antimicrobial therapy was started with cefepime and amphotericin B deoxycholate. Blood culture resulted positive for *Pseudomonas aeruginosa* and cefepime was switched to imipenem. After one week of this regimen, patient was still neutropenic, febrile and worsening clinical conditions. A new set of blood cultures was positive for *Fusarium* sp. The skin biopsy showed a large number of hyphae using direct microscopic exam, morphologically compatible with fusariosis. The patient died in November due to refractory septic shock.

### *Fusarium*samples

Four clinical isolates of *Fusarium* sp. from clinical specimens were identified as: LIF 2008, 2009 and 2010 recovered from blood cultures (BacT/ALERT® 3D, bioMérieux AS, France), LIF 1994 from skin biopsy and F111 from the air of the patient’s hospital room.

## Microorganism identification

The five clinical isolates were cultured in Sabouraud Dextrose agar (Difco, Sparks, Maryland, USA) and identified by morphologic criteria after subculture by macroscopic and microscopic characteristics (Verweij et al. 2007).

## Molecular methods

DNA extraction of *Fusarium* spp. from blood culture bottles, from skin biopsy and from air were performed using Dr. GenTLE® kit (Takara, Otusu, Shiga, Japan). After DNA measurement in NanoDrop 2000 (Thermo Scientific, Wilmington, USA) and equalization to a concentration of 2 ng/μL, DNA samples were analyzed using: DNA microarray and DNA sequencing.

The DNA microarray was performed as described by (Ferrari et al. 2013), the oligonucleotide probes, consisting of 14 to 20 species-specific nucleotide sequences with biotin-labeled poly T anchors at the end of each nucleotide (Invitrogen, Showajima, Japan), were designed based on ITS1 and ITS2 sequences of the Type strains [GenBank database, American Type Culture Collection (ATCC), Centraalbureau voor Schimmelcultures (CBS) and MMRC-Chiba (IFM)]. Multiple-sequence alignments were performed using the BioEdit software (version 7.1.3. [http://www.mbio.ncsu.edu/BioEdit/bioedit.html]). Conserved regions were also used as targets for genus-specific probes or as controls. The probe sequences were spotted onto a plastic slide (NGK Insulators LTD, Aichi, Japan) using a KCS mini microarray printer (Kubota Comps. Corporation, Amagasaki, Japan). For fungal identification, PCR using universal fungal primers ITS1 (5′-TCCGTAGGTGAACCTGCGG-3′) and ITS4 (5′-TCCTCCGCTTATTGATATGC-3′) (Sigma-Aldrich, Saint Louis, USA) were used to amplify the ITS regions (ITS1 and ITS2) and the 5.8S rRNA gene followed by hybridization, conjugation, coloration and direct visualization of specifically positioned spots on the slide that were consisted of 23 fungal species.

The sequencing was performed as described before (Moreira-Oliveira et al. 2005), with modifications. First, we performed a polymerase chain reaction (PCR) to amplify a region of the gene *EF1α* using the pairs of primers forward HS392 (5′-TCAAAATGGGTAAGGA(A/G)GACAAGAC-3′) and HS393 (5′-GCCTGGGA(G/A)GTACCAGT(G/C)ATCATGTT-3′). The PCR products were directly sequenced with a BigDye© terminator reagent kit (Applied Biosystems, Foster City, CA) in an automated DNA sequencer (3110 AB System, Applied Biosystems, Foster City, USA). Besides HS392 and HS393 primers, two internal initiators were used for sequencing reaction and amplification of *EF1α* gene: EF11 (5′-GTGGGGCATTTACCCCGCC-3′) and EF21 (5′-GAGTGGCGGGGTAAATGCC-3′). The sequences were assembled using ATSQ version 6.0.1 (Genetix) and compared with database information available at NCBI bank (National Center for Biotechnology Information, http://blast.ncbi.nlm.nih.gov/Blast.cgi). Sequences alignment was carried out in Clustal Omega (http://www.ebi.ac.uk/Tools/msa/clustalo/). The sequencing was performed in the Molecular Epidemiology Laboratory at the Faculty of Medical Sciences, State University of Campinas, São Paulo, Brazil and the Medical Mycology Research Center, Chiba University, Chiba, Japan.

## Antifungal susceptibility test

Antifungal susceptibility tests were performed for samples LIF 2008, LIF 2009, LIF 2010, 1994 and EF111. Minimal inhibitory Concentrations (MIC) and minimal effective concentration (MEC) were determined following the micro dilution method recommended by CLSI document M38-A2, with minor changes. The conidia forms were suspended by gently probing the colony with a sterile Pasteur pipette tip to dislodge the conidia from the hyphal mat and the solution; then, they were counted in a Neubauer chamber and adjusted to a suspension containing 4 × 10^4^ conidia/mL (CLSI 2008; Teixeira et al. 2005). After that, conidia were re-suspended in RPMI 1640 (Sigma) with L-glutamine, without sodium bicarbonate, and buffered with 0.165 mol/L 3-morpholinopropanesulfonic acid (MOPS) in pH 7.0. The final suspension was distributed in microdilution plates containing pre-defined incremental concentrations of amphotericin B (AMB); itraconazole (ITZ); voriconazole (VOR); micafungin (MCF); 5-flucytosine (5FC); miconazole (MCZ) and fluconazole (FCZ). The plates were incubated at 35°C and fungal growth was eye-observed after 24 h and 48 h. The MIC was defined as the lowest drug concentration that caused 100% of inhibition of visible fungal growth for AMB, ITZ and VCZ and as the lowest drug concentration that caused 50% of inhibition of visible fungal growth for MCZ, 5FC and FCZ and the MEC for MCZ was defined as the lowest concentration of drug that leads to the growth of small, rounded, compact hyphal forms as compared to the hyphal growth seen in the growth control well. The tests were performed in duplicate. American Type Culture Collection strains *Candida parapsilosis* ATCC 22019, *Candida krusei* ATCC 6258, *Candida albicans* ATCC 76615 and *Candida albicans* ATCC 90028 were used for quality control.

### Antifungal agents

Micafungin, amphotericin B, 5-flucytosine, fluconazole, voriconazole, itraconazole and miconazole in pre-prepared dry plates (Eiken Chemical Co., Ltd., Tokyo) (Makimura et al. 2005).

## Results

### Assessment of the *Fusarium napiforme*strains

**Figure 1:**
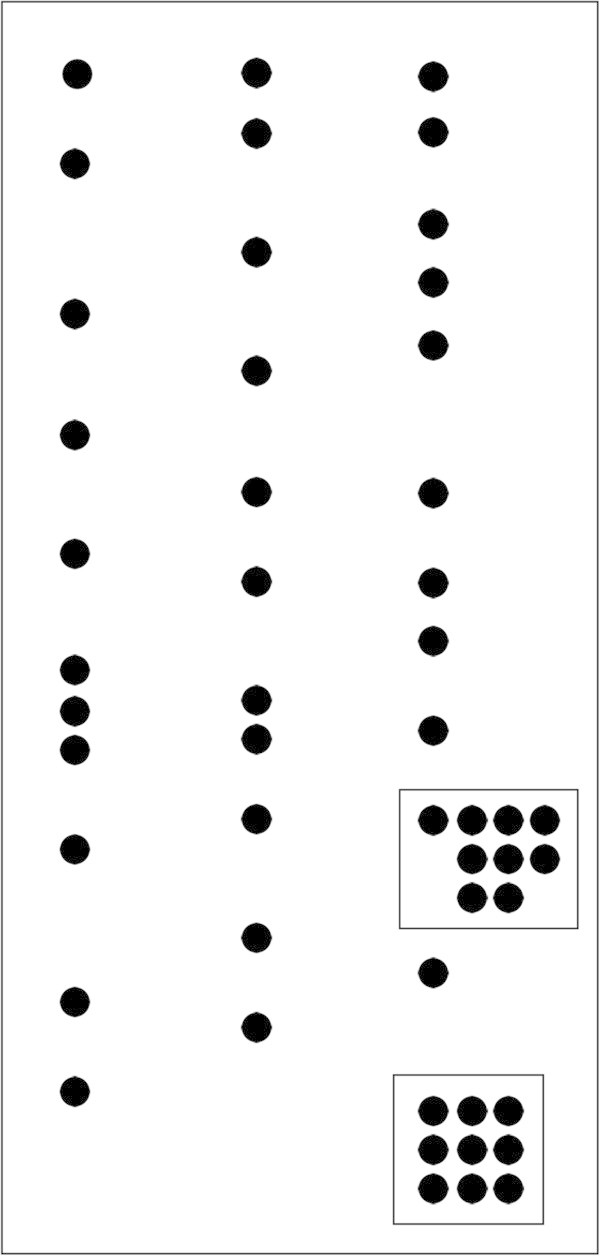
**Representative hybridization pattern of** ***Fusarium*** **non-** ***solani*** **species complex and positive control using DNA microarray.** A group of specific hybridization spots are visualized inside the square above and the spots remaining are representative of biotin (negative for others species). The positive control is shown inside the below square and represents a sequence common to all fungal species.

**Figure 2:**
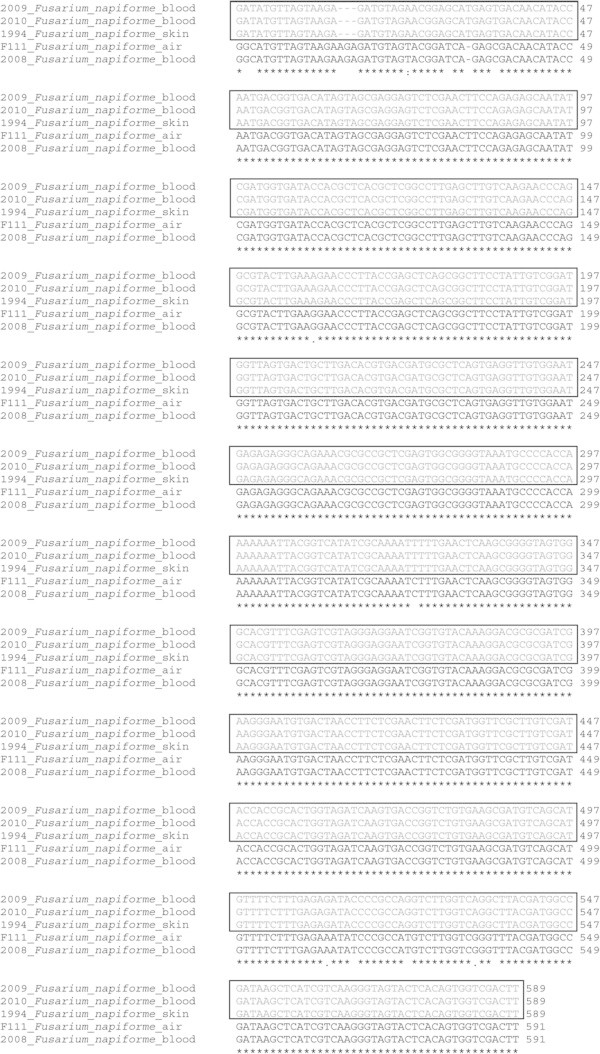
**Clustal Omega multiple sequences alignment of** ***Fusarium napiforme*** **isolates by** ***EF1α*** **gene sequencing.** The * symbol indicate a 100% homology for the specific base position. The square indicates one of the genetic variant groups.

All isolates from blood, skin biopsy and from air were identified as *Fusarium* sp. by macroscopic and microscopic characteristics, as *Fusarium* non-*solani* species complex (FNSSC) DNA microarray methodology (Figure 1) and as *F. napiforme* (non-*solani*) by sequencing (Table 1). The sequences alignment showed two *F. napiforme* distinct groups based on DNA homology: one group with *F. napiforme* samples isolated from blood (2009, 2010) and from skin biopsy (1994), and another containing isolates for blood (2008) and air (F111), as shown in Figure 2.

**Table 1:** **Results of identification of Fusarium species according macro- and micro- morphological characteristics, DNA microarray and DNA sequencing**

Isolate LIF	Macro- and micro-morphological characteristics	DNA microarray	DNA sequencing	% Similarity*
2008	*Fusarium* sp.	FNSSC	*Fusarium napiforme*	98
2009	*Fusarium* sp.	FNSSC	*Fusarium napiforme*	99
2010	*Fusarium* sp.	FNSSC	*Fusarium napiforme*	99
1994	*Fusarium* sp.	FNSSC	*Fusarium napiforme*	99
F111	*Fusarium* sp.	FNSSC	*Fusarium napiforme*	98

LIF = Fungal Research Laboratory, FNSSC *= Fusarium* non-*solani* species complex, *Similarity % for the comparison with NCBI database.

The susceptibility results can be seen in Table 2.

**Table 2:** **Literature review of antifungal drugs evaluated for** ***Fusarium*** **species**

		Antifungal agent μg/ml (MEC/MIC)*						
Author [reference]	***Fusarium***species	AMB	FLU	MCF	ITZ	VOR	5FC	MCZ
(Melcher et al. 1993)	*Fusarium napiforme*	1.16	10		1.25		>322.7	
(Durand-Joly et al. 2003)	*Fusarium solani*	1						
(Rodriguez et al. 2003 )	*Fusarium oxysporum*	2				4		
(Rothe et al. 2004)	*Fusarium oxysporum*	2	>64		<2		>64	
(Guzman-Cottrill et al. 2004)	*Fusarium solani*	8	>64			8	>64	
(Teixeira et al. 2005)	*Fusarium solani*	2			128			
(Ho et al. 2007)	*Fusarium solani*	1			>16	4		
(Neuburger et al. 2008)	*Fusarium proliferatum*	4-8	>16		>16	8		
(Tortorano et al. 2008)	*Fusarium verticillioides*	1.53			3.33	1.74		
	*Fusarium solani*	1.25			>16	9.21		
	*Fusarium proliferatum*	1.7			>16	4.2		
	*Fusarium oxysporum*	2.3			>16	4		
	*Fusarium subglutinans*	3.3			10.8	5.6		
(Xie et al. 2008)	*Fusarium solani*							8
(Tezcan et al. 2009)	*Fusarium verticillioides*	1-2			>8	4		
(Bose et al. 2011)	*Fusarium* spp.	2-4						
(Liu et al. 2011)	*Fusarium solani*	1				4		
(Sekeroglu et al. 2012)	*Fusarium solani*	0,5	>64			8		
(Pereira et al. 2013)	*Fusarium solani*	>8			>8	0.5-0.128		
	*Fusarium oxysporum*	≤2			8			
(Fanci et al. 2013)	*Fusarium verticillioides*	2				8		
(Inano et al. 2013)	*Fusarium solani*		>4	>16	>8	8		16
	*Fusarium moniliforme*							8
	*Fusarium oxysporum*							8
Present study								
isolate 2008	*Fusarium napiforme*	2-4	1-2	>16	>8	>8	>64	0.125-0.25
isolate 2009	*Fusarium napiforme*	2-4	1-2	>16	>8	2	>64	0.125-0.25
isolate 2010	*Fusarium napiforme*	2-4	1-2	>16	>8	4	>64	0.125-0.25
isolate 1994	*Fusarium napiforme*	2	1-2	>16	. > 8	4	>64	0.125
isolate F111	*Fusarium napiforme*	1	8	>16	>8	2	>64	0.5

*MEC = minimal effective concentration, MIC = minimum inhibitory concentration, MEC was defined for micafungin and MIC for the other drugs, AMB = amphotericin B, FLU = fluconazole, MCF = micafungin, ITZ = itraconazole, VOR = voriconazole, 5FC = 5-flucytosine, MCZ = miconazole.

## Discussion

*Fusarium* spp. are emerging as pathogens that can cause life-threatening invasive opportunistic infections, mainly among patients with bone marrow suppression and neutropenia. Currently, *Fusarium* spp. are considered the second most-common mold as cause of opportunistic infection in these patients, being *Aspergillus* spp. the first ones (Bodey et al. 2002; Cooke et al. 2009). *Fusarium* spp. are also the most common cause of fungemia with skin manifestations. (Bodey et al. 2002) reported 76% of 46 patients with hematologic malignancies or solid tumors, considered to have definite *Fusarium* infections, had skin lesions. (Nucci and Anaissie 2007) also reported 61 hematopoietic stem cell transplant recipients with hematologic malignancies or solid tumors with disseminated *Fusarium* infection, and metastatic skin lesions was the most frequent clinical presentation, occurring in 46(75%) of patients.

The combination of cutaneous lesion and positive blood cultures, involving or not other sites are the most frequent pattern of disseminated fusariosis. The most common clinical presentation is a persistently febrile patient with prolonged and profound neutropenia who develops disseminated characteristic skin lesions, with a positive blood culture for a filamentous fungi (Nucci and Anaissie 2007). This was the case of our patient.

The most common *Fusarium* involved in human infections are *F.solani, F.oxysporum* and *F.verticillioides* (Gupta et al. 2000; Tezcan et al. 2009). To our knowledge, we describe here the second case of disseminated fusariosis caused by *F.napiforme*, and the first case with identification confirmed by molecular techniques, including sequencing.

The first invasive case by *F.napiforme* was described in 1993 (Melcher et al. 1993) in a patient diagnosed with acute myeloid leukemia, under cytoreduction and profound granulocytopenia following high-dose of cytosine arabinoside, mitoxantrone, and VP-16. Since then, no other cases have been described.

The diagnosis of *Fusarium* in laboratory includes some criteria such a positive direct mycological examination showing typical septated hyphae branching at 45°. However, the identification of *Fusarium* to the species level is often difficult and requires a specialized laboratory and skilled personnel. In such situations, molecular biology techniques might be helpful for the definitive diagnosis. Furthermore, the early diagnosis of invasive disease might be helpful to guide the correct antifungal therapy, which is crucial for patient recovery (Galimberti et al. 2012; Busemann et al. 2009; Azor et al. 2009). In our study, molecular methods allowed a faster and accurate identification of the causative agent as belonging to *F.napiforme* group. In addition, the strains F111 and 2008, from blood and air respectively, showed 100% sequencing alignment, suggesting that the the air may have been the source of the infection. Samples 2009 and 2010, isolated from the blood, and skin isolate 1994 were also aligned. Thus, it seems that we had two variants of *F.napiforme*. Therapy for invasive fusariosis is a challenging problem, mainly because *Fusarium* shows high MICs to antifungal agents, and therefore, there is no proven effective treatment regimen (Tezcan et al. 2009; Guzman-Cottrill et al. 2004; Rothe et al. 2004). The treatment of choice for invasive fusariosis is amphotericin B. However, it is controversial, since there are reports of *Fusarium* showing MICs for AMB ranging from 1 to 4 μg/mL. Triazoles, as voriconazole and posaconazole, have also been used successfully (Pereira et al. 2013; Tortorano et al. 2008). Furthermore, different *Fusarium* can exhibit variable susceptibility patterns.

In the present case, patient was treated with amphotericin B deoxicolate, initiated after laboratory confirmation of *Fusarium* fungemia. However, the time between the onset of symptoms and the blood culture was fifteen days and the time between the blood culture and the positive result for *Fusarium* was fifteen days more. The isolate of *F.napiforme* in the present case was resistant to amphotericin B, with MIC ranging from 2-4 μg/ml. Delay in antifungal therapy plus the resistance profile could have contributed to the patient’s death three days after antifungal drug was initiated.

In conclusion, *Fusarium* spp. are emerging as a fungi of relevance in medical mycology, since they are associated with low susceptibility profiles to antifungal drugs and high mortality rate, mainly in imunocompromissed patients. These facts highlight the importance for faster and more accurate diagnostic tests, contributing to earlier and precise diagnosis and treatment of this life-threatening infection.

### Nucleotide sequence accession numbers

The sequences determined in this study have been submitted in (Sakai et al. 2014) and deposited in NCBI database with the accession numbers KM099396 to KM099400.

### Consent

Informed consent was obtained from the patient for the publication of this report and any accompanying images.
